# Immune correlates of metastatic melanoma patients treated with ipilimumab in combination with fotemustine in the phase II NIBIT-M1 study

**DOI:** 10.1186/2051-1426-1-S1-P107

**Published:** 2013-11-07

**Authors:** Cristina Maccalli, Hugues Nicolay, Filippo Capocefalo, Ester Fonsatti, Carla Chiarucci, Ornella Cutaia, Diana Giannarelli, Giorgio Parmiani, Anna Maria Di Giacomo, Michele Maio

**Affiliations:** 1 Unit of Immuno-biotherapy of Melanoma and Solid Tumors, Fondazione Centro San Raffaele, Milan, Italy; 2 Division of Medical Oncology and Immunotherapy, Azienda Ospedaliera Universitaria, Senese, Istituto Toscano Tumori, Siena, Italy; 3 Statistics, Regina Elena National Cancer Institute, Rome, Italy; 4 On behalf of the NIBIT-M1 Investigators group, Siena, Italy

## Background

Ipilimumab (IPI) in combination with fotemustine (FTM) has shown a promising clinical activity in metastatic melanoma (MM) patients (pts) enrolled in the NIBIT-M1 trial (Di Giacomo, et al., Lancet Oncology, 2012). This study investigated changes in immunological parameters in the course of treatment.

## Material and methods

MM pts received an induction therapy with IPI 10 mg/kg every 3 weeks (Q3W) for four doses and FTM 100 mg/m2 weekly for 3 weeks. Peripheral blood lymphocytes (PBMC) and sera were collected at baseline, wk12, and wk24 to perform phenotypic and functional T cell assays, and to investigate humoral responses against a panel of tumor-associated antigens (TAAs) and soluble NKG2D ligands (sNKG2DL).

## Results

Circulating central memory T (Tcm) cell populations, both CD4+ and CD8+, co-expressing CD45RO, CD27, CD28, CCR7, CD62L, were increased following treatment both at wk12 and wk24. Interestingly, T cells co-expressing CD4, BTLA and CD45RO were increased in pts with clinical benefit, while CD8+ Tcm co-expressing BTLA were augmented in pts with objective responses. Circulating T cells reactive against NY-ESO-1, MART-1, gp100 and TYRP-1, were found in 12/23 pts expressing at least one of the HLA-A1, A2, -A3 or A24 alleles with induction or augmentation of TAA reactivity in the course of treatment. Moreover, at least one sNKG2DL was found, though heterogeneously, in sera of 26/38 pts. Of note, in 10 pts, down-modulation of at least 2/4 investigated sNKG2DLs were detectable in relation with TAA-specific responses. Finally, at baseline, antibodies against NY-ESO-1, MAGE-A3, SSX-2, HMW-MAA, TYRP-1 and MSLN were found in sera of 9-15-8-7-7-6 of 40 investigated MM pts, respectively. Substantial changes were seen during therapy showing induced humoral responses against at least one investigated TAA in 10/40 pts at wk12 and/or wk24. In addition, up-regulation, equivalent to at least two-fold of the pre-existing antibody levels, against at least one TAA was detected in 5/40 pts at wk12 and/or wk24.

## Conclusions

Our results, although preliminary, indicate that IPI in combination with FTM in MM pts induced changes in circulating T subpopulations and in their TAA-specific responses as well as in circulating antibodies to selected TAAs probably contributing to the observed clinical activity.

